# The impact of lymphovascular space invasion on survival in early stage low-grade endometrioid endometrial cancer

**DOI:** 10.1186/s40001-023-01084-9

**Published:** 2023-03-13

**Authors:** Fariba Yarandi, Elham Shirali, Setare Akhavan, Fatemeh Nili, Sara Ramhormozian

**Affiliations:** 1 Department of Gynecologic Oncology, Yas Hospital, Tehran University of Medical Sciences, Tehran, Iran; 2 Department of Gynecologic Oncology, Vali-Asr Hospital, Tehran University of Medical Sciences, Imam Khomeini Hospital Complex (IKHC), Tehran, Iran; 3 Department of Pathology, Tehran University of Medical Sciences, Imam Khomeini Hospital Complex, Tehran, Iran

**Keywords:** LVSI, Endometrial cancer, Staging, Survival, Prognostic factor, Low-grade endometrioid adenocarcinoma

## Abstract

**Background:**

The lymphovascular space invasion (LVSI) is suggested as a prognostic factor for endometrial cancer in many studies, but it has not yet been employed in FIGO staging system. The present study was aimed to evaluate the impact of LVSI on survival in patients with early stage endometrioid endometrial cancer.

**Methods:**

This retrospective cohort was conducted on early stage endometrial cancer patients who underwent surgical staging [total abdominal hysterectomy and bilateral salpingo-oophorectomy (TAH/BSO)] and omental biopsy at Referral Teaching Hospitals of Tehran from 2005 to 2021. Patient’s age, menopause status, tumor grade, tumor size, depth of myometrial invasion, LVSI and lower segment involvement were recorded. Data were analyzed with SPSS 22.

**Results:**

415 patients with stage I and grade 1–2, endometrioid endometrial cancer were analyzed. 100 patients (24.1%) were LVSI-positive. 3-year and 5-year survival rates were 97.1% and 88.9%, respectively. Recurrence occurred in 53 patients (12.8%). 3-year overall survival rates in LVSI-negative and LVSI-positive were 98.7% and 92%. These rates for 5-year survival were 92.1% and 79%, respectively. Recurrence rates in LVSI-negative were 8.9% while it was 25% in LVSI-positive cases. Multivariate analysis showed that LVSI has significant correlation with 3-year and 5-year overall survival rates.

**Conclusions:**

LVSI in early stage endometrial cancer significantly and independently influences 3-year and 5-year survival rates and acts as a strong prognostic factor in these patients. LVSI should be implemented in endometrial cancer staging systems due to its significant correlation with cancer recurrence rates and 5-year survival rates.

## Background

Endometrial cancer (EC) is the most common gynecologic malignancy with over 65000 estimated new cases at 2022 in the United States [1]. Staging of endometrial cancer is performed by surgical methods and majority of patients are diagnosed in early stages with 5-year overall survival of over 80% [1]. Stage is considered the most important prognostic factor in these patients but other factors including histologic grade, depth of invasion, tumor size, age and lymphovascular space invasion (LVSI) also contribute to patients’ prognosis [2]. “The Cancer Genome Atlas (TCGA)” project [38] also led to description of the molecular EC classification. In the years after the publication of TCGA’s results, several studies were able to identify four subgroups analogous to those described by the use of surrogate markers: *POLE*-ultramutated (*POLE*mut), mismatch repair deficient (MMRd), p53-abnormal (p53abn) and No Specific Molecular Subgroup (NSMP) [37]. The molecular EC classification with this approach has proven to have a strong prognostic value in clinical trials and unselected cohorts [39–43].

LVSI is pathologically defined as detection of tumoral cells in lymphatics or small vessels outside the core tumor. LVSI is reported to be found in up to 35% of patients with endometrial cancer [3]. Numerous studies have revealed that LVSI is associated with lower overall survival, higher risk of recurrence, lymph node metastasis and distant metastasis [2, 4–9]. Studies have claimed that LVSI correlates with poorer prognosis in endometrial cancers of FIGO stage I–III [10].

Standard treatment of early stage endometrial cancer is total hysterectomy, bilateral salpingo-oophorectomy (BSO) and dissection of pelvic lymph nodes with or without dissection of para-aortic lymph nodes [4]. Complete lymphadenectomies for staging harbor high burden of morbidities which impair patients’ quality of life including development of lymphedema, lymphocele and neuralgia [11, 12]. It is also observed that complete lymphadenectomy does not yield benefits for survival [13, 14].

In an attempt to avoid the disadvantages of lymphadenectomy, the use of sentinel lymph node (SLN) biopsy has been proposed as an alternative staging procedure with increasing evidence that SLN mapping is efficient in identifying metastatic nodal disease without compromising oncological safety [47]. When optimal technique and careful intraoperative nodal assessment are applied, achieving comparable or even superior detection rates to those of lymphadenectomy could be accessible with this emerging technique; however, future studies are still needed to safely replace “complete lymphadenectomy” since there are potential areas of doubt and debate, especially for high-risk endometrial cancer cases which should be resolved [46].

In line with the considerations previously mentioned, efforts are made to lower the rates of lymphadenectomy for staging. Thus, pathologic criteria have been proposed to omit lymphadenectomy in patients with enough low risk of recurrence. For instance, Mayo criteria have defined a group of patients with grade I/II tumors, endometrioid histology, less than 50% myometrial invasion and tumor size of less than 2 cm who do not require lymphadenectomy. Relatively similar criteria have also been published by European Society of Medical Oncology and Korean GOG [15–18]. Due to elective lymphadenectomy procedure, patients with LVSI in final pathology are found who are NX (The regional lymph nodes have not been evaluated). In addition, there are patients who have indications for lymphadenectomy, but do not undergo this procedure at surgery because of incidental finding of undiagnosed cancer during operation, medical comorbidities or patient’s unwillingness.

As it is well known, identification and risk stratification of patients with endometrial cancer is so crucial for their treatment and prognosis. Given the prognostic implications of LVSI in the studies, NCCN, GOG-99 and ESGO guidelines have used LVSI as a factor for classification of endometrial cancer patients into low-, intermediate or high-risk groups, but FIGO has not still employed LVSI as one of the components of its staging system. So, we aimed to evaluate the impact of LVSI on patients’ survival specifically in low-risk group defined as stage I, grade 1–2 endometrioid endometrial cancer.

## Methods

This experimental retrospective cohort was conducted at referral teaching hospitals of Tehran from 2005 to 2021. All of the endometrial cancer cases were evaluated, but the study sample was confined to stage I (FIGO 2018 staging system), grade 1–2 endometrioid endometrial cancer based on final hysterectomy specimens who had undergone surgical staging by total abdominal hysterectomy with bilateral salpingo-oophorectomy (BSO) and omental biopsy. To further increase the accuracy of results and decrease the interobserver bias, all of the pathology reports were reassessed by a qualified gynecologic pathologist before final analysis. Also, dissection of pelvic and para-aortic lymph nodes was performed in majority of cases. Factors including age, menopause status, parity, comorbidities, pathological findings (tumor grade and size, depth of myometrial invasion, LVSI and lower segment involvement) and adjuvant therapy if performed, were recorded. Hormone receptor status was also evaluated in a few number of patients. LVSI was defined as presence of viable tumoral cells in channels covered by lymphatic or vascular endothelial epithelium outside the tumoral mass. For histopathologic evaluation of LVSI, presence of cohesive tumor clusters with smooth borders conforming to the shape of a lymphvascular space, in the invasive border of the tumor, in hematoxylin and eosin-stained sections was assessed [45]. Disaggregated tumor cells associated with cell debris and inflammatory cells differing in shape and size from the harboring vessel, were considered artifacts [45]. Patients with tumoral emboli in the spaces covered by endothelial epithelium were also classified as LVSI positive. Immigration of malignant cells into endothelium in intra-endothelial vascular channels was considered as a necessary step for progression of metastatic disease. Overall survival (OS) defined as the length of time being alive from surgical staging till death, was considered as the primary endpoint of the study. The study was approved by ethics review board of Tehran University of Medical Sciences (TUMS).

SPSS 22 software was used for data analysis. For descriptive analysis of data, frequency and percentage or mean and standard deviation were used. For comparison of quantitative variables in different groups, ANOVA test was used. Post hoc analysis was also used for pairwise comparison of means or percentages. To compare categorical parameters between groups, Chi-square test was also used. Statistical significance was considered as *p*-value < 0.05.

## Results

689 cases of endometrial cancer were reviewed. 415 patients (60.23%) had endometrioid endometrial cancers in stages IA and IB with grade I–II. Other cases were excluded from the analysis due to higher stages or pathologies other than endometrioid endometrial cancer. Mean age of patients was 54.53 ± 9.63 years. 272 patients (65.5%) were in post-menopausal status. The most common comorbidities were diabetes mellitus (131 patients, 31.6%) and hypertension (55 patients, 13.3%). 316 patients (76.1%) were in stage IA while 99 patients (23.9%) were in stage IB. Histologic grades were also I and II for 269 (64.8%) and 146 (35.2%) patients, respectively.

100 patients (24.1%) were LVSI-positive in our study. Recurrence had also occurred in 53 patients (12.8%). 3-year and 5-year overall survival rates were 97.1% (403 patients) and 88.9% (369 patients). Recurrence occurred in 28 LVSI-negative (8.9%) patients while this rate in LVSI-positive group was as high as 25% (25 patients). 3-year overall survival rates for LVSI-negative and LVSI-positive groups were 98.7% and 92%, respectively (*p* = 0.000). These rates for 5-year overall survival rates were 92.1% and 79%, respectively (*p* = 0.000). The details of study parameters in both LVSI-negative and LVSI-positive groups are presented in Table 1.

**Table 1:** Study parameters in LVSI-negative and LVSI-positive groups

Parameter	LVSI-negative	LVSI-positive	*p*-value
Age (years)	54.46 ± 9.57	54.77 ± 9.88	0.782
Parity	3.55 ± 2.67	3.29 ± 2.52	0.402
Menopause status	203 (64.4%)	69 (69%)	0.620
Chief complaint at diagnosis			
Post-menopausal bleeding	201 (64.2%)	68 (68%)	0.444
Menometrorrhagia	106 (33.9%)	31 (31%)	0.197
Past medical history	155 (63.5%)	36 (62.1%)	0.836
Comorbidity			
Diabetes mellitus	85 (37.6%)	46 (59.7%)	0.025
Hypertension	47 (20.8%)	8 (10.4%)	
Hypothyroidism	9 (4%)	5 (6.5%)	
Hypertension and diabetes mellitus	47 (20.8%)	12 (15.6%)	
Diabetes mellitus and hypothyroidism	7 (3.1%)	1 (1.3%)	
Hypertension and hypothyroidism	9 (4%)	1 (1.3%)	
Diabetes mellitus, hypertension and hypothyroidism	7 (3.1%)	0 (0%)	
Tumor size (cm)	3.21 ± 1.81	3.67 ± 1.95	0.040
D&C pathology			
Low-grade endometrioid carcinoma	261 (84.7%)	81 (81%)	0.329
Endometrioid carcinoma/hyperplasia with atypia	34 (11%)	9 (9%)	
Well-differentiated endometrioid carcinoma on polyp	4 (1.3%)	3 (3%)	
Final pathology			
Endometrioid carcinoma	303 (96.2%)	96 (96%)	0.524
Synchronous ovarian/uterine endometrioid carcinoma	1 (0.3%)	0 (0%)	
Endometrioid carcinoma on polyp	3 (1%)	2 (2%)	
Myometrial invasion			
< 50%	222 (70.5%)	53 (53%)	0.000
> 50%	56 (17.8%)	45 (45%)	
Absence of myometrial invasion	36 (11.4%)	1 (1%)	
Lower segment involvement	46 (14.6%)	23 (23%)	0.049
Adjuvant therapy			
No adjuvant therapy	170 (54.7%)	30 (30%)	0.000
Brachytherapy	98 (31.5%)	31 (31%)	
Brachytherapy and EBRT^a^	38 (12.2%)	34 (34%)	
Stage			
IA	260 (82.5%)	56 (56%)	0.000
IB	55 (17.5%)	44 (44%)	
Grade			
I	213 (67.6%)	56 (56%)	0.034
II	102 (32.4%)	44 (44%)	
Lymphadenectomy			
No lymphadenectomy	121 (38.5%)	40 (40%)	0.954
Only pelvic lymphadenectomy	158 (50.3%)	49 (49%)	
Sampling(suspicious lymph nodes)	19 (6.1%)	5 (5%)	
Complete (pelvic and para-aortic lymphadenectomy)	16 (5.1%)	6 (6%)	
Recurrence	28 (8.9%)	25 (25%)	0.000
Recurrence site			
Pelvis	9 (34.6%)	10 (40%)	0.847
Abdomen	8 (30.8%)	6 (24%)	
Distant	7 (26.9%)	8 (32%)	
Pelvis and distant	1 (3.8%)	1 (4%)	
Cervix	1 (3.8%)	0 (0%)	
Recurrence time (months)	33.65 ± 21.02	23.04 ± 14.88	0.043
Overall 3-year survival	311 (98.7%)	92 (92%)	0.000
Overall 5-year survival	290 (92.1%)	79 (79%)	0.000

^a^*EBRT* external beam radiotherapy

Given the importance of adjuvant therapy in patients’ survival, 3-year and 5-year survival rates were compared according to adjuvant therapy type and LVSI status. Despite this analysis showing significant difference between survival rates in different adjuvant therapy groups, this impact on survival did not remain significant on multivariate regression analysis. The details of comparison of various adjuvant therapies according to LVSI status are presented in Table 2.

**Table 2:** Survival rates based on adjuvant therapies in LVSI-negative and LVSI-positive groups

Parameter	Survival period	LVSI-positive	LVSI-negative	*p*-value
No adjuvant therapy	3 years	90%	98.2%	0.044
	5 years	80%	93.6%	0.025
Brachytherapy	3 years	93.3%	100%	0.013
	5 years	77.4%	91.8%	0.037
Brachytherapy and EBRT	3 years	97.1%	100%	0.472
	5 years	79.4%	92.1%	0.112

To determine the factors independently and significantly influencing survival rates, univariate analysis was performed. Tables 3, 4 present factors significantly impacting 3-year and 5-year overall survival rates in univariate analysis.

**Table 3:** Factors with significant impact on 3-year survival rate in univariate analysis

Parameter	Non-survivors	Survivors	*p*-value
Positive LVSI	8 (66.7%)	92 (22.8%)	0.000
Adjuvant therapy			
No adjuvant therapy	6 (50%)	195 (48.8%)	0.012
Brachytherapy	3 (25%)	126 (31.5%)	
Brachytherapy and EBRT	1 (8.3%)	71 (17.8%)	
Recurrence	9 (75%)	44 (10.9%)	0.000
Recurrence time	9.22 ± 8.28	32.57 ± 17.94	0.000

**Table 4:** Factors with significant impact on 5-year survival rate in univariate analysis

Parameter	Non-survivors	Survivors	*p*-value
Parity	4.51 ± 3.56	3.37 ± 2.49	0.011
LVSI	21 (45.7%)	79 (21.4%)	0.000
Recurrence	21 (45.7%)	32 (8.7%)	0.000
Recurrence site			
Pelvis	2 (9.5%)	17 (56.7%)	0.001
Abdomen	6 (28.6%)	8 (26.7%)	
Distant	11 (52.4%)	4 (13.3%)	
Pelvis and distant	2 (9.5%)	0 (0%)	
Recurrence time	21.95 ± 18.49	33.00 ± 18.05	0.038

Factors in Tables 3, 4 were then analyzed with multivariate regression analysis to omit the effect of confounding variables and determine the factors significantly and independently influencing survival. The results of multivariate regression analysis revealed that only LVSI was a significant predictor of 3-year and 5-year overall survival.

It must be noted that the exact data on the time of death in our patients were lacking because we only checked the status of alive/dead in 3-year and 5-year time-points. The data presented in the tables are based on the status of patients’ survival in these two time-points. Binary logistic regression was used for the analysis with a binary variable (Yes/No) of being alive at 3 and 5 years as the dependant variable. The parameters used in the regression analysis included all the variables of study through a stepwise selection strategy (starting with forward selection).

## Discussion

In this study, we aimed to evaluate the impact of LVSI on the survival rates in patients with early stage low-grade endometrioid endometrial cancer. We found out that LVSI is significantly correlated with 3-year and 5-year overall survival rates. 100 patients (24.1%) were LVSI-positive in our study. In line with our study, Akhavan et al. [19] have also reported a rate of 23.8% for LVSI positivity in endometrial cancer patients. Various reports have revealed rates from 8.3 to 32.8% for LVSI-positive cases [19–28]. These wide variations in rates of LVSI-positive cases in studies might be attributed to different study designs, LVSI definitions and study populations in terms of stage and grade. Another explanation can be that in patients with apparently low-risk endometrioid endometrial cancer with low clinical stage (IA or IB), patients do not undergo lymphadenectomy due to related risks. In these patients, frozen section is requested and LVSI detection on frozen section specimens could be very difficult and challenging in many situations. Thus, LVSI is rarely reported on frozen sections. Essentially LVSI negativity on frozen sections does not preclude presence of LVSI. So, some studies might have used frozen section specimens which leads to lower reported rates of LVSI.

Overall recurrence rate was 12.8% in our study which was 8.9% and 25% in LVSI-negative and LVSI-positive cases, respectively. Not only recurrence rate, but also recurrence time were significantly correlated with LVSI. Ureyen et al. [20] has reported that recurrence occurred in 3.4% of cases. Multivariate analysis in their study showed that LVSI was significantly associated with recurrence which was consistent with our study. Restaino et al. [21] also reported that recurrence rates in LVSI-negative, focal-LVSI and diffuse-LVSI cases were 6.6%, 14.7% and 24.9% which were quite similar to our findings. That study also showed that LVSI was significantly and independently associated with recurrence and distant metastasis. An analysis of endometrioid-type endometrial carcinoma in the PORTEC-1 and -2 trials showed that the three-tiered LVSI scoring system (none versus focal versus substantial) had the highest predictive values for regional recurrence and distant metastasis. Substantial LVSI compared with none or focal LVSI is a prognostic factor for pelvic recurrence, distant metastasis and overall survival [44]. There was no difference in recurrence site and treatment type according to LVSI groups in our study. Thus, risk of pelvic and extra-pelvic recurrence was not different based on presence or absence of LVSI.

LVSI was significantly associated with higher rates of diabetes mellitus, larger size of tumor, more extensive myometrial invasion, more involvement of lower segment, higher rates of adjuvant therapy and higher stages which all can contribute to worsening of disease control and patients’ survival. Tortorella et al. [3] have reported that diffuse LVSI was significantly correlated with histologic grade, higher myometrial infiltration and higher tumor size. Patients with LVSI had higher chance of need for adjuvant therapy (6.6% versus 52.6%). Dai et al. [24] also reported that diabetes history, lymph node metastasis, deep myometrial invasion and absence of progesterone receptor (PR) expression were independently correlated with LVSI. These findings all support the prognostic role of LVSI in endometrial cancer patients.

Overall 3-year and 5-year survival rates were 97.1% and 88.9% in our study. Presence of LVSI decreased 3-year survival from 98.7 to 92% and 5-year survival from 92.1 to 79% which both were significant changes. Multivariate regression analysis showed that LVSI was significant predictor of 3-year and 5-year survival. LVSI was the only factor significantly influencing both 3-year and 5-year survival rates. This finding underscores the importance of LVSI as a prognostic factor in early stage low-grade endometrioid endometrial cancer patients. Ureyen et al. [20] showed that disease-free survival was decreased from 96.8 to 80.1% in LVSI-positive cases. They also reported that LVSI and tumor grade were significantly associated with disease-free survival. Tortorella et al. [3] also showed that 5-year survival was decreased from 99.5 to 70.6% in patients with positive LVSI. Disease-free survival also showed a decline from 93.6 to 56.5% with presence of LVSI. They claimed that LVSI is the strongest predictor of poor survival in endometrial cancer patients. While many studies support our findings, few studies exist which reject the prognostic role of LVSI in endometrial cancer patients. For instance, Askandar et al. [29] reported that overall survival rates in LVSI-positive and LVSI-negative patients were 50% and 55.3% which do not show significant difference. They also revealed that LVSI did not have any significant prognostic impact on overall survival in multivariate analysis. Dai et al. [24] also reported that multivariate analysis showed no additional risk for patients in presence of LVSI.

Overall, based on the current and previous findings, LVSI is available as a strong prognostic factor. In addition, LVSI status is important in decision-making for lymphadenectomy. Studies have shown that lymphadenectomy is associated with higher morbidity and no improvement of survival in low-risk patients [30–32] while survival rate increases with lymphadenectomy in moderate- and high-risk patients [33, 34]. Patients with moderate and high levels of risk benefit from pelvic and para-aortic lymphadenectomy. Thus, it is necessary to define low, moderate and high-risk groups. It seems that LVSI can be a key factor in differentiating low- and moderate-risk patients. In stage IA (G1 and G2) with endometrioid histology, LVSI status can be determining for endometrial cancer risk stratification. Based on ESMO modified classification, patients can be categorized as low-risk without LVSI and presence of LVSI changes the class to moderate-risk group [35]. Our study also showed that due to significant decline of survival in patients with LVSI, risk of LVSI-positive and LVSI-negative patients is not the same and they should be classified in separate groups. Balaya et al. [36] conducted a study on 2018 FIGO IB stage patients and they showed that in patients with the same stage, LVSI decreases disease-free survival significantly from 95.8 to 82.5%. They concluded that LVSI must be employed in staging of early stage endometrial cancer patients.

There exist some limitations in our study, the main being absence of data on the duration between treatment and death of patients which limited the possibility of more comprehensive evaluation of survival status. Another limitation relates to the absence of the molecular EC classification for most of our patients, so it was not assessed. Evaluation and reporting the extent of LVSI (focal versus extensive) which is a new concept was also not reported in many previous pathology reports. Due to limited access to the old pathology slides and paraffin blocks, review of the slides was not possible as well. All of these could be intriguing topics of research for future studies.

## Conclusions

Overall, we can conclude that LVSI in early stage low-grade endometrioid endometrial cancer significantly and independently influences 3-year and 5-year survival rates and acts as a strong prognostic factor in these low-risk patients. Also, size of tumor and myometrial invasion impact 3-year and 5-year survival rates, respectively. Due to higher risk of recurrence and lower risk of survival even in low-grade endometrioid endometrial cancer patients in presence of LVSI, it seems that LVSI should be implemented in endometrial cancer staging systems in the future.

## Data Availability

All data generated or analyzed during this study are available for review by the Editor-in-Chief of this journal on request.

## Abbreviations

LVSI: Lymphovascular space invasion
FIGO: The International Federation of Gynecology and Obstetrics
TAH: Total abdominal hysterectomy
BSO: Bilateral salpingo-oophorectomy
OS: Overall survival
ESGO: European Society of Gynecological Oncology
NCCN: The National Comprehensive Cancer Network
